# Longitudinal study of *Clostridium difficile* shedding in raccoons on swine farms and conservation areas in Ontario, Canada

**DOI:** 10.1186/s12917-015-0563-x

**Published:** 2015-10-07

**Authors:** Kristin J. Bondo, J. Scott Weese, Joyce Rouseau, Claire M. Jardine

**Affiliations:** Department of Pathobiology, University of Guelph, Guelph, Ontario, N1G 2W1 Canada; Department of Pathobiology and Canadian Wildlife Health Cooperative, University of Guelph, Guelph, ON N1G 2W1 Canada

**Keywords:** *Clostridium difficile*, Conservation area, Longitudinal study, *Procyon lotor*, Raccoon, Swine farm

## Abstract

**Background:**

*Clostridium difficile* is an important enteropathogen affecting humans, domestic animals, and wildlife. The objectives of this study were to 1) compare the prevalence and characteristics of *C. difficile* isolated from the feces of raccoons trapped on swine farms and conservation sites, and 2) investigate the role of raccoons as potential reservoirs for host-adapted strains of *C. difficile* using a longitudinal study. Fecal swabs were collected from raccoons at 5 conservation sites and 5 swine farms, once every five weeks, from May to November, 2012.

**Results:**

*Clostridium difficile* was isolated from 9 % (38/444) of samples, from 12 % (37/302) of raccoons, from all 10 sites. A total of 19 different ribotypes were identified, including 5 ribotypes that matched recognized international designations and which are also found in humans (001, 014, 056, 078, and 103). Location type (farm or conservation area) was not associated with *C. difficile* status (*P* = 0.448) but only 3 ribotypes (014, 056, and 078) were found in both location types. The prevalence of ribotype 078 was significantly higher on farms (4 %; 9/220) compared to conservation sites (1 %; 2/225) (*P* = 0.034). Only one of 108 raccoons caught in multiple sessions was positive on more than one occasion.

**Conclusions:**

We found no evidence to support the hypothesis that raccoons harbour host-adapted strains of *C. difficile*; rather, it appears that raccoons transiently acquire *C. difficile* from the environment. Raccoons are unlikely to be maintaining *C. difficile*, but because we detected *C. difficile* strains that have the potential to cause illness in humans and livestock, and because raccoons can move relatively large distances, they may play a role in the dissemination of pathogenic ribotypes of *C. difficile* throughout the environment.

## Background

*Clostridium difficile* is an anaerobic bacterium that is an important enteropathogen in humans and some domestic animals [1, 2]. *Clostridium difficle* is one of the top 5 infectious causes of human mortality in Ontario, responsible for 327 deaths annually [3]. In addition to humans and domestic animals, disease associated with *C. difficile* has been reported in a variety of wildlife species [4, 5]. *Clostridium difficile* can also be found in soil and water and in the intestinal tracts of apparently healthy humans and animals, including wildlife [6–8]. Although wild animals are reservoirs for various infectious agents and can be involved in the transmission of pathogens to and from humans and domestic animals [9], the role of wildlife, if any, in the epidemiology of *C. difficile* is not clear [10].

Raccoons are commonly found in close association with humans and domestic animals. They are known to harbour a number of zoonotic pathogens, including *C. difficile* and in a previous study of raccoons trapped on farms, four unique *C. difficile* isolates were identified*,* none of which were present in the authors’ reference library of over 3,000 human and domestic animal *C. difficile* isolates [10]. Based on these findings, we hypothesized that raccoons may carry host-adapted strains of *C. difficile* that are unrelated to direct or indirect (e.g., environmental) exposure from humans or domestic animals. The objectives of this study were to 1) compare the prevalence and characteristics of *C. difficile* isolated from the feces of raccoons living on swine farms and conservation areas (areas with no exposure to livestock), 2) assess the impact of location type (farm versus conservation area), raccoon demographic factors, and season on the prevalence of *C. difficile* in raccoons, and 3) investigate the role of raccoons as potential maintenance hosts of host adapted strains of *C. difficile* using a longitudinal study.

## Methods

Procedures for trapping and handling animals were approved by the Animal Care Committee at the University of Guelph following the guidelines of the Canadian Committee on Animal Care. Raccoons were live-trapped on 5 swine farms and 5 conservation areas in southern Ontario within a 100-km radius of either Guelph (43°32’42” N 80°15’01” W) or Cambridge (43°21’49” N 80°18’50” W) Ontario from May to October in 2012. Distance between sites ranged from 1.3 to 52.2 km.

At each site, 20**–**40 Tomahawk live traps were set for 3–4 nights once every 4-5 weeks (Sizes 106 and 108; Tomahawk Live Trap Co. Tomahawk, Wisconsin, USA). Captured raccoons were brought to a centralized holding area for processing unless they had already been caught that week, in which case they were released immediately. Raccoons were anesthetized using an intramuscular injection of 0.025 mg/kg dexmedetomidine hydrochloride (Dexdomitor 0.5 mg/ml; Pfizer Animal Health, Kirkland, Quebec, Canada) and 5 mg/kg ketamine hydrochloride (Vetalar 100 mg/ml; Bioniche Animal Health, Belleville, ON, Canada) before being removed from the trap. A numbered metal ear tag (1005-3, National Band and Tag Co. Newport, Kentucky, USA) was placed in one ear and a passive integrated transponder tag (GPT12 Pre-Load Sterile; Biomark, Boise, Idaho, USA) was injected subcutaneously between the shoulder blades for subsequent identification. Sex, age class (adult or juvenile, on the basis of animal size and teeth wear and staining), and mass were recorded for each animal. Fecal swabs were collected per rectum using Cary-Blair applicators (BBL CultureSwab, Becton, Dickinson and Company, Annapolis, Maryland, USA) and then refrigerated.

To isolate *C. difficile*, swabs were immersed in *C. difficile* moxalactam-norfloxacin (CDMN) broth (Oxoid Ltd., Nepean, ON, Canada) with 0.1 % taurocholate and incubated anaerobically at 37 °C. After 7 days, alcohol shock was performed by mixing 2 ml of broth with 2 ml absolute ethanol. After 1 h incubation at room temperature, samples were centrifuged and the pellet was inoculated onto CDMN agar for anaerobic incubation for 48 h. *Clostridium difficile* was tentatively identified by colony morphology, Gram stain appearance and l-proline aminopeptidase production. A single isolate from each sample was characterized by ribotyping [11], PCR detection of tcdA, tcdB and cdtA/cdtB [12], and toxinotyping [13]. Isolates that visually matched ribotype patterns of reference strains from the Cardiff/ECDC reference collection were assigned the corresponding designation (e.g., 078). Otherwise, isolates were compared to an internal collection.

Logistic regression models with random effects were constructed in STATA (STATA Intercooled 13.1; StataCorp, College Station, Texas, USA) to examine associations between the presence of *C. difficile* and four variables: location type (conservation area or swine farm), season (late May–July or August–October), age (juvenile or adult), and sex (male or female). Two distinct seasons were considered: rearing (May–July) and pre-denning/dispersal (August–October) as defined by Rosatte et al. (2010) [14]. Random effects for site and animal were included to account for autocorrelation among samples taken from the same site and the same animal. Univariable models, i.e., one fixed effect per model, were initially created with random effects and then multivariable models that included interaction terms, main and random effects were built. In creating multivariable models, a main effects model was initially constructed, variables that were not statistically significant were removed assuming they were not potential confounding variables, and then all possible interactions were individually examined. Variables were retained in the final model if they were significant, part of a significant interaction term or acted as a confounding variable. A variable was considered to be a confounding variable if it was a nonintervening variable and its removal from the model resulted in ≥ 30 % change in any of the coefficients of a statistically significant variable [15]. Variance partition coefficients (VPCs) were estimated from the variance components of the final model including both fixed and random effects using the latent variable technique [15]. Random effects were excluded from models if their inclusion explained very little of the variation and if excluding them resulted in a model with a better fit or no change based on Akaike’s Information Criterion (AIC) and Bayesian Information Criterion (BIC) [15]. Odds ratio and 95 % confidence interval (CI) of each variable were reported.

Due to small sample sizes, exact logistic regression was used to compare the prevalence of specific ribotypes of *C. difficile* between the conservation and farms sites. Associations for all statistical tests were considered significant at α = 0.05.

## Results

*Clostridium difficile* was isolated from 8.6 % (95 % CI, 6.1–11.6 %; 38/444) of samples from 12.3 % (95 % CI, 8.8–16.5 %; 37/302) of raccoons from all 10 locations. Only one of 108 raccoons caught in multiple sessions was positive at more than one time. Samples from this raccoon were collected 8 weeks apart, and different ribotypes were found at each sampling period. Of the 108 recaptures, 83 occurred in consecutive months. Seventy–two of these raccoons were negative on all captures, 3 were negative on one occurrence and positive on subsequent capture (4-5 weeks later), and 8 were positive on one occurrence and negative upon a subsequent capture. One adult male raccoon was caught at two different swine farms (1.3 km apart), so one of the results from this animal was randomly removed from the statistical analysis.

In the univariable models, *C. difficile* was more likely to be detected in raccoons sampled in May–July than in August–October and more likely to be detected in adult than juvenile raccoons (Table 2). The final model included only season because in the multivariable logistic regression model, season remained significant (OR = 3.78; 95 % CI, 0.094–0.74, *P* = 0.011), but age was no longer significant because season confounded age (OR = 2.55; 95 % CI, 1.36–10.58, *P* = 0.231). In addition, when the other variables were included together in the model with season, none of the other variables were confounding variables, and there were no significant interactions. Based on the estimates of the VPCs, site and sample level (i.e., swab tested) explained 9.1 % and 90.9 %, respectively, of the variance in *C. difficile* occurrence in the model with season (Table 2).

Of the 38 isolates, 20 (52.6 %; 95 % CI, 35.8–69.0 %) had *tcdA* and *tcB,* but not *cdtA*, 14 (36.8 %; 95 % CI, 21.8–54.0 %) had all three genes and 4 (10.5 %; 95 % CI, 2.9–24.8 %) had no toxin genes (Table 1). A total of 19 different ribotypes were identified, including 5 ribotypes that matched recognized international designations, 4 that matched previously identified ribotypes in our laboratory (including four that have been previously identified in humans and 1 that was detected in a raccoon in 2010), and 10 that have not been previously identified in our laboratory. Overall, 28/34 (82 %; 95 % CI, 65.5–93.2 %) toxigenic isolates were ribotypes that have been previously identified in humans by this laboratory while 6/34 (18 %; 95 % CI, 6.8–34.5 %) were strains that have only been identified by this laboratory in raccoons.

**Table 1:** Relative frequencies of *Clostridium difficile* ribotypes (with toxin profile and toxinotype) isolated from raccoons trapped on farms and conservation areas in Ontario, Canada

Ribotype^a^	Toxin gene(s)	Toxinotype	No. (%) of isolates from farms *n* = 219	No. (%) of isolates conservation from areas *n* = 225	Total no. (%) isolates *n* = 444
001	A+B+CDT-	0	0	2 (0.9 %)	2 (0.4 %)
014	A+B+CDT-	0	2 (0.9 %)	4 (1.8 %)	6 (1.3 %)
056	A+B+CDT-	XII	2 (0.9 %)	1 (0.4 %)	3 (0.7 %)
078	A+B+CDT+	V	9 (4.1 %)	2 (0.9 %)	11 (2.5 %)
103	A+B+CDT-	II	0	1 (0.4 %)	1 (0.2 %)
AG	A+B+CDT-	0	0	1 (0.4 %)	1 (0.2 %)
F	A+B+CDT-	0	1 (0.5 %)	0	1 (0.2 %)
I	A+B+CDT-	0	0	2 (0.9 %)	2 (0.4 %)
O	A+B+CDT-	0	0	1 (0.4 %)	1 (0.2 %)
WR10	A-B-CDT-	NA	1 (0.5 %)	0	1 (0.2 %)
WR12	A+B+CDT+	V	0	1 (0.4 %)	1 (0.2 %)
WR13	A+B+CDT-	0	1 (0.5 %)	0	1 (0.2 %)
WR3	A+B+CDT+	IV	1 (0.5 %)	0	1 (0.2 %)
WR4	A+B+CDT+	III	1 (0.5 %)	0	1 (0.2 %)
WR5	A+B+CDT-	0	1 (0.5 %)	0	1 (0.2 %)
WR6	A-B-CDT-	NA	1 (0.5 %)	0	1 (0.2 %)
WR7	A-B-CDT-	NA	0	1 (0.4 %)	1 (0.2 %)
WR8	A-B-CDT-	NA	1 (0.5 %)	0	1 (0.2 %)
WR9	A+B+CDT-	0	1 (0.5 %)	0	1 (0.2 %)

^a^Numerical identifiers match international designations, letter identifiers were assigned to ribotypes previously detected in our laboratory, and WR numbers were assigned to ribotypes that have not been previously identified in our laboratory or that have only been see in wildlife (i.e., WR3 which was detected in a raccoon in 2010)

Location type (farm or conservation area) was not associated with *C. difficile* status (*P* = 0.448; Table 2), but 9/19 ribotypes were found only on farms and 7/19 ribotypes were found only in conservation areas (Table 1). Only 3 ribotypes (014, 056, and 078) were found in both location types (Table 1). The prevalence of ribotype 078 was significantly higher on farms (9/219) compared to conservation sites (2/225) (OR = 4.8; 95 % CI, 0.97–45.8; *P* = 0.034), but there was no significant difference in prevalence of ribotype 056 (OR = 2.1; 95 % CI, 0.11–122.3; *P* = 0.619) or ribotype 014 (OR = 51; 95 % CI, 0.04–3.6; *P* = 0.686) between location types.

**Table 2:** Univariable logistic regression model with random effects (site and animal) showing associations between *Clostridium difficile* status and raccoon age (adult or juvenile), sex, location type (farm or conservation area), and season (May to July or August to October)

Predictor	Sub-category (*n* = sample size)	% *C. difficile* positive (95 % CI)^a^	Univariable models^b^				
			Odds ratio	95 % CI	Covariance parameter estimates (95 % CI)		*P*
					Site level	Animal level^d^	
Age^c^	Juvenile (*n* = 83)	2.4 (0.3–8.4)	Referent				
	**Adult (** ***n*** **= 360)**	10.0 (7.1**–**13.6)	**4.79**	**1.11–20.60**	**0.28 (0.04–1.8)**		**0.036**
Location type	Conservation (*n* = 225)	7.1 (4.1–11.3)	Referent				
	Swine farm (*n* = 219)	10.0 (6.4–14.8)	1.42	0.57**–**3.59	0.21 (0.02–1.8)		0.448
Sex	Female (*n* = 245)	8.6 (5.4–12.8)	Referent				
	Male (*n* = 199)	8.5 (5.1–13.3)	0.93	0.46**–**1.88	0.27 (0.04–1.9)	0.14^e^	0.831
Season	Aug. to Oct. (*n* = 165)	3.0 (1.0–6.9)	Referent				
	**May to July (** ***n*** **= 279)**	11.8 (8.3–16.2)	**4.81**	**1.80–12.87**	**0.21 (0.06–1.8)**		**0.002**

^a^CI = confidence interval

^b^Significant differences are in bold

^c^Age for 1 individual was unknown

^d^The random effect for animal was not included in the models for age, location type, and season because it explained 5.3 × 10^-31^ to 4.2 × 10^-30^ of the variance, and its removal from the model changed the coefficients little and did not change the model according to AIC and BIC

^e^The 95 % confidence interval was estimated to be between 3.6 × 10^-11^ and 5.6 × 10^8^

## Discussion

The prevalence of *C. difficile* in fecal samples from raccoons in this study (9 %) was similar to what was detected in a previous study of raccoons in Ontario (8 %) [10]; however, we detected *C. difficile* in raccoons at all sites sampled in this more recent study and only at 16 % of farm sites in 2010. We also detected a greater variety of ribotypes, including ribotypes that are known to be associated with human and livestock disease, compared to the previous study by Jardine et al. (2013) [10]. The greater sample size used in the current study may be responsible for the observed higher site prevalence and greater ribotype diversity of *C. difficile*, but it is also possible that different ribotypes have emerged in raccoons since 2010 [10].

In contrast to the previous study by Jardine et al. (2013) [10], five internationally recognized *C. difficile* ribotypes that are known to be associated with disease in humans and/or livestock were detected in raccoons in this study. These included ribotypes 001, 014, and 078, which were the top three ribotypes detected in samples from hospitalized patients in the Netherlands in 2009 [16] and among the top 11 ribotypes detected in symptomatic patients in North America [17]. Although these ribotypes are also commonly detected in livestock and environmental samples [18, 19] transmission between humans, animals and the environment has not been proven [20]. The emergence of ribotype 078, a strain over-represented in community-associated disease in humans in some regions, has been epidemiologically linked to its occurrence in livestock, suggesting that there is at least the potential for zoonotic transmission [20]. The detection of known pathogenic ribotypes of *C. difficile* and isolates with toxin genes *tcdA* and *tcB,* which are known to be associated with human disease, in raccoons raises concerns about their potential to act as a component of the *C. difficile* reservoir and contribute to the transmission of *C. difficile* to humans [21].

In our longitudinal study, only one of 108 raccoons caught on multiple occasions tested positive repeatedly for *C. difficile,* and this individual had different strains of *C. difficile* at each trapping session. We therefore conclude that *C. difficile* shedding in raccoon feces is transient with raccoons harbouring *C. difficile* for only short periods of time (<5 weeks). A similar pattern of transient *C. difficile* shedding was described in a longitudinal study of healthy horses [22] and dogs [23]. Our conclusions are subject to a couple of caveats. First, because we only tested a single *C. difficile* colony per sample, we could not determine if raccoons were simultaneously colonized with multiple ribotypes. Second, it is not clear if our findings represent intermittent true colonization or detection of ingested spores transiently passing through the intestinal tract [24]. Although further work is required to clarify these aspects of *C. difficile* shedding in raccoons, it seems clear that raccoons are unlikely to be maintaining *C. difficile* for any length of time. Although the prevalence of *C. difficile* in raccoons did not differ between location type (farm or conservation area), there was little overlap in strain types between farms and conservation areas. In addition, although ribotype 078 was found on both farms and conservation areas, it was significantly more likely to occur in raccoons trapped on farms than conservation areas. This, combined with the high prevalence of ribotype 078 in some livestock farms [25], suggests that raccoons obtain *C. difficile* as a consequence of environmental exposure and may, therefore, act as sentinels for *C. difficile* in the environment. We were not able to obtain samples from farm animals or the environment for *C. difficile* testing in this study and were unable to factor clustering by site in the statistical analyses comparing prevalence of specific ribotypes between location types because of small effective sample size. Because three of the farms were in close proximity to one another, they may not have been entirely independent units. Further studies with larger sample sizes and more study sites that concurrently examine the occurrence of *C. difficile* in wildlife, livestock and the environment will help to identify potential sources of *C. difficile* for wildlife.

Based on the univariable models, the prevalence of *C. difficile* in raccoons was higher from May to July than from August to October and higher in adults than juveniles; however, in the multivariable model, season confounded age. In humans, *Clostridium difficile* infections occur more frequently in the winter months [26]; however, we did not sample raccoons in the winter in this study. Himsworth et al. (2014) [27] found no association between season and *C. difficile* status in wild urban rats (*Rattus* spp). Young age, particularly prior to weaning, is associated with *C. difficile* shedding in several species [28, 29]. Because all of the raccoons in our study were free-ranging and live-trapped, juveniles we captured were at least in the process of being weaned. In our study, most adults (72 %; 261/360) were captured during May to July whereas most juveniles (80 %; 66/83) were captured August to November. This disproportionate number of adults and juveniles captured according to season may have influenced the relationship between age and season in the multivariable model. Additional studies, occurring over longer time periods, with larger sample sizes, and with raccoons prior to weaning are required to better understand the seasonal and demographic factors that may be associated with *C. difficile* occurrence in wildlife.

## Conclusions

We found no evidence to support the hypothesis that raccoons are maintaining host-adapted strains of *C. difficile*; rather, it appears that raccoons transiently acquire *C. difficile* from the environment. We detected *C. difficile* strains in raccoons that have the potential to cause illness in humans and livestock; however, all raccoons sampled for this study appeared clinically normal and there was no apparent impact of *C. difficile* shedding on raccoon health. Although raccoons are unlikely to be maintaining *C. difficile*, they have been observed to move as far as 45 km in Ontario [14] and may therefore play a role in the dissemination of pathogenic ribotypes of *C. difficile* throughout the environment.
